# Gene expression analysis supports tumor threshold over 2.0 cm for T-category breast cancer

**DOI:** 10.1186/s13637-015-0034-5

**Published:** 2016-02-08

**Authors:** Hiroko K. Solvang, Arnoldo Frigessi, Fateme Kaveh, Margit L. H. Riis, Torben Lüders, Ida R. K. Bukholm, Vessela N. Kristensen, Bettina K. Andreassen

**Affiliations:** 1 Department of Marine Mammals, Institute of Marine Research, C. Sundts Gate 64, Bergen, 5004 Norway; 2 Department of Biostatistics, Institute of Basic Medical Science, University of Oslo, Norway and Statistics for Innovation—(sfi)2, Oslo, Norway; 3 Medical Genetics Department, Oslo University Hospital (Ullevål), Oslo, Norway; 4 Department of Surgery, Akershus University Hospital, Lørenskog, Norway; 5 Department of Molecular Biology and Laboratory Sciences (EpiGen), Institute of Clinical Medicine, Akershus University Hospital, Lørenskog, Norway; 6 Institute of Clinical Medicine, University of Oslo, Norwegian Center of HPH Network, Oslo, Norway; 7 Department of Genetics, Institute for Cancer Research, Oslo University Hospital Radiumhospitalet, Oslo, Norway; 8 Cancer Registry of Norway Institute for Population-based Research, Oslo, Norway

**Keywords:** Breast cancer, T-category, Differentially expressed, Microarray data, Two-group comparison statistical test, Optimization algorithm

## Abstract

Tumor size, as indicated by the T-category, is known as a strong prognostic indicator for breast cancer. It is common practice to distinguish the T1 and T2 groups at a tumor size of 2.0 cm. We investigated the 2.0-cm rule from a new point of view. Here, we try to find the optimal threshold based on the differences between the gene expression profiles of the T1 and T2 groups (as defined by the threshold). We developed a numerical algorithm to measure the overall differential gene expression between patients with smaller tumors and those with larger tumors among multiple expression datasets from different studies. We confirmed the performance of the proposed algorithm by a simulation study and then applied it to three different studies conducted at two Norwegian hospitals. We found that the maximum difference in gene expression is obtained at a threshold of 2.2–2.4 cm, and we confirmed that the optimum threshold was over 2.0 cm, as indicated by a validation study using five publicly available expression datasets. Furthermore, we observed a significant differentiation between the two threshold groups in terms of time to local recurrence for the Norwegian datasets. In addition, we performed an associated network and canonical pathway analyses for the genes differentially expressed between tumors below and above the given thresholds, 2.0 and 2.4 cm, using the Norwegian datasets. The associated network function illustrated a cellular assembly of the genes for the 2.0-cm threshold: an energy production for the 2.4-cm threshold and an enrichment in lipid metabolism based on the genes in the intersection for the 2.0- and 2.4-cm thresholds.

**Electronic supplementary material:**

The online version of this article (doi:10.1186/s13637-015-0034-5) contains supplementary material, which is available to authorized users.

## Introduction

Breast cancer is known as a complex biological system, and tumors are complex organ systems shaped by gene aberrations, cellular biological context, characteristics specific to the person, and environmental factors. Management of breast cancer relies on the availability of robust clinical and pathological prognostic and predictive factors to guide patient decision-making and the selection of treatment options [1]. Tumor size, indicated by the T-category, is known as a strong prognostic indicator for breast cancer and is one of the factors taken into account when deciding how and whether to treat a patient, independent of lymph node status. Significantly better survival can be expected in tumors categorized as T1. It is common practice to distinguish between T1 (0.1 cm < and < 2.0 cm) and T2 (2.0 cm < and < 5.0 cm) groups by the 2-cm rule [1]. It is well known that the T1-T2 distinction is reflected in prognosis: tumors categorized into the T2 group are more aggressive and might have already spread.

Gene expression profiling has in the last decade entered the field of molecular classification. An array-based approach to characterize T1 and T2 tumors was recently attempted, based on microarray data that present the expression level for each feature (gene or probe) and revealed distinct molecular pathways characterizing each stage [2]. The differential expression (DE) for a feature is measured using two-group comparison, for which several statistical methods, such as *t*-statistics, significant analysis of microarray (SAM), fold changes, and *B*-statistics, have been proposed [3]. However, DE measures are obviously dependent on the threshold chosen to distinguish between T1 and T2 tumors. In fact, the study by Riis et al. [2] suggested that using the T-size expression signatures instead of tumor size leads to a significant difference in risk for distant metastases and that the molecular signature can be used to select patients with tumor category T1 who may need more aggressive treatment and patients with tumor category T2 who may have less benefit from it. To stratify patients into two groups each requiring a different treatment for breast cancer, ‘Cutoff Finder’ was developed by [4]. The ‘Cutoff’ point is determined by the distribution of the marker under investigation and optimizing the correlation of the dichotomization with regard to an outcome or survival variable. The method was considered for stratifications based on the expression of specific genes, estrogen receptor, and progesterone receptor, neither whole genomic regions nor tumor size. In this article, we develop an algorithm to evaluate the traditional 2.0-cm threshold in the light of gene expression differences between breast cancer patients below and above the threshold. We use two different measurements from meta-analysis theory that are useful for handling multiple genetic studies; these apply different pre-processing techniques, platforms, and lab environments. The choice of which meta-analysis technique to use depends on the type of response and objective. When the objective is to identify the DE between two conditions, methods include vote counting, combining ranks, *p* values, and effect sizes [3]. Campain and Yang provided an intuitive measure, called meta differential expression via distance synthesis (mDEDS) [5], using DE via distance synthesis (DEDS) [6] to aggregate multiple DE measurements. The performance of mDEDS was compared with existing meta-analysis methods, such as Fisher’s inverse chi-square, GeneMeta, metaArray, RankProd, and Naïve meta-methods, using a simulation study and two case studies [3]. The results mostly showed better performance for mDEDS, while some cases favored the Fisher’s inverse chi-square [7]. This method uses a simple procedure that combines the *p* values from independent datasets. Therefore, we apply both the mDEDS and the Fisher’s score in our proposed algorithm in order to analyze different thresholds. To confirm the reliability of the proposed algorithm, we performed a simulation study. Then, we applied this algorithm to three different expression datasets gathered at two Norwegian hospitals. To validate the estimated optimum threshold for the Norwegian datasets, we applied our algorithm to five publicly available expression datasets. Based on the estimated optimum threshold for the Norwegian datasets, we investigated the prognostic status from the viewpoints of local recurrence and the associated network and canonical pathway.

## Method

Given *i* = 1, ⋅ ⋅⋅, *I* genes from *k* = 1, ⋅ ⋅⋅, *K* datasets, the measures are described below. We should use two measures of comparison.

### Fisher’s inverse chi-square statistic

Let *p*_*ik*_ indicate the *p* value obtained by a DE statistic for the *i*th gene and *k*th dataset. The Fisher summary statistic *S*_*i*_ [6] for each gene *i* is defined as

1 $$ {S}_i=-2{\displaystyle {\sum}_{k=1}^K \log \left({p}_{ik}\right)} $$

This statistic tests the null hypothesis that gene *i* is not the DE between the two groups given *K* datasets. Under this null hypothesis, *S*_*i*_ is chi-square distributed with 2*K* degrees of freedom. In our case, the *p* value is calculated by the Wilcoxon-Mann-Whitney (WMW) test for each gene and each dataset.

### Differential expression via distance synthesis (DEDS)

It is possible to calculate various statistics to describe the differences in expression between the two groups, including WMW test, *t*-statistics, and fold change (FC). DEDS then integrates and summarizes these statistics using a weighted distance approach [6] used for two-group comparisons, and next, it measures the distance between the aggregated point and the extreme origin that is assumed to represent the largest measurement of all. These procedures can be performed by the R package called ‘*DEDS*’ (http://www.bioconductor.org/). In the procedure, *t*-, SAM, FC, *B*-, moderated *t*-, and moderated *F*-statistics were selected as *t*_*j*_. Campain and Yang expanded DEDS to a meta-analysis method, called mDEDS [5]. The flow for the analysis by mDEDS proceeds as follows. (1) Apply *J* appropriate statistics *t*_*ij*_ to each of *i* = 1, ⋅ ⋅⋅, *I* genes and *J* with 1 ≤ *J* ≤ 6. The observed coordinate-wise extreme point over all genes is defined by *E*_0_ = (max_*i*_(*t*_*i*1_), ⋅ ⋅⋅, max_*i*_(*t*_*iJ*_)). (2) For each permuted dataset *b* = 1, ⋅ ⋅⋅, *B*, obtain the permutation extreme point *E*_*b*_ and evaluate the coordinate-wise extreme point *E*_*p*_ by maximizing over all permutations *E*_*p*_ = (max_*b*_(*E*_*b*1_), ⋅ ⋅⋅, max_*b*_(*E*_*bJ*_)). (3) Obtain the overall maximum *E* = max(*E*_0_, *E*_*p*_). (4) Calculate the distance *d*_*i*_ from each gene to *E* = (*E*_1_, ⋅ ⋅⋅, *E*_*J*_), defined by ${d}_i={\displaystyle {\sum}_{j=1}^J\frac{{\left({t}_{ij}-{E}_j\right)}^2}{\mathrm{MAD}{\left({t}_{ij}\right)}^2}}$, where MAD is the median absolute deviation from the median. (5) Do steps (1)–(4) for all *k* = 1, ⋅ ⋅⋅, *K* studies and summarize the distances coordinate-wise. The package outputs the list for estimated statistics and the distance for each dataset. To perform procedure (5), we summarize the obtained distances for all datasets and order them according to the genes.

### An extension to DEDS

For mDEDS, the original study [5] did not touch on the procedure for using the extreme origin to measure the distance between the points by applying measurements that may change across different cohorts. DEDS’s original procedure selects the larger one of the original data or the permutated data as the extreme origin, obtained without taking into account changes in the extreme origin. In fact, the extreme origin and the coordinate-wise extreme origin changed if the dataset changed. When mDEDS is calculated for the threshold shifting at 0.1 intervals within a region from 1.5 to 3.5, the origin should also change in this manner: ${E}_{1.5}= \max \left({E}_0^{(1.5)},{E}_p^{(1.5)}\right)$ for *q* = 1.5,…, ${E}_q= \max \left({E}_0^{(q)},{E}_p^{(q)}\right)$ for *q*,…, ${E}_{3.5}= \max \left({E}_0^{(3.5)},{E}_p^{(3.5)}\right)$ for *q* = 3.5, where ${E}_0^q$ and ${E}_p^q$ indicate the extreme point obtained by the original data and permuted data, respectively. Therefore, we define the following extreme point, named ‘totally extreme point (TEP)’: *E*_max_ = max(*E*_1.5_, ⋅ ⋅⋅, *E*_*q*_, ⋅ ⋅⋅, *E*_3.5_) if *q* ∈ (1.5, 3.5)_._

Then, the scaled distance for each gene across studies *K* is ${d}_i={\displaystyle {\sum}_{k=1}^K{\displaystyle {\sum}_{j=1}^6\frac{{\left({t}_{ikj}-{E}_{\max}\right)}^2}{\mathrm{MAD}{\left({t}_{ikj}\right)}^2}}}$.

### Estimation of optimal threshold *q* between T1 and T2

Our intention is to identify the optimal threshold used to divide the sample into two groups, such that it best distinguishes the differential expression pattern between these two groups. To identify this threshold, we define the following optimization problem for an optimal threshold *q*_0_ within a set *Q* of candidate thresholds. Let *S*_*i*_(*q*) be the Fisher score (1) applied to the two group comparison using a threshold at *q*, i.e., ${S}_i(q)={\displaystyle {\sum}_{k=1}^K{p}_{ik}(q)}$. Then

2 $$ {q}_{0\_\mathrm{Fisher}}=\underset{q\in Q}{ \arg \max }{\displaystyle {\sum}_{i=1}^I{S}_i(q)}\kern0.5em \mathrm{f}\mathrm{o}\mathrm{r}\kern0.5em \mathrm{Fisher}\hbox{'}\mathrm{s}\kern0.5em \mathrm{s}\mathrm{core} $$

and similarly for

3 $$ {q_0}_{\_\mathrm{mDEDS}}=\underset{q\in Q}{ \arg \min }{\displaystyle {\sum}_{i=1}^I{d}_i(q)}\kern0.5em \mathrm{f}\mathrm{o}\mathrm{r}\kern0.5em \mathrm{mDEDS} $$

For the TEP introduced in 2.3, we take the summation of the distance for all genes and estimate the threshold that minimizes this value as

4 $$ {q}_{0-\mathrm{T}\mathrm{E}\mathrm{P}}=\underset{q\in Q}{ \arg \min }{\displaystyle {\sum}_{i=1}^I{d}_i} $$

This is motivated by the idea that we are looking for the threshold that best divides the two tumor groups from each other based on the genome-wide expression profiles.

**Fig. 1:**
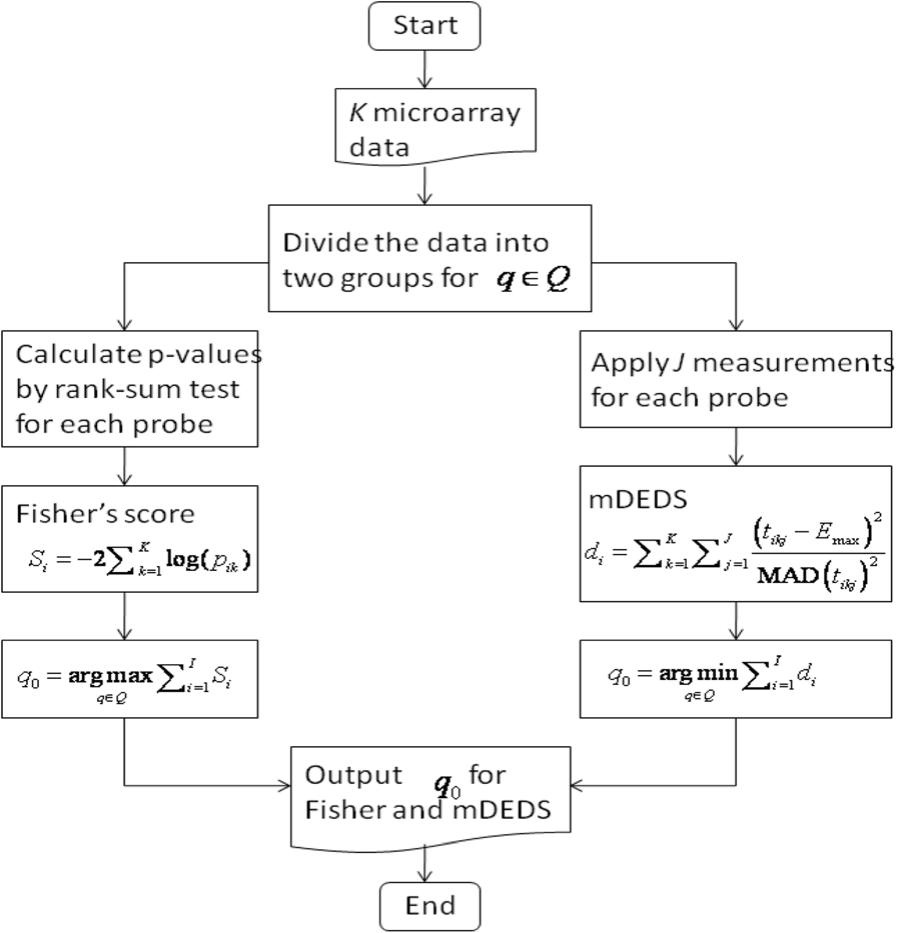
Flow diagram of the analysis process

For possible thresholds *q* in *Q*, we evaluated the Fisher’s score and mDEDS values. A flow chart of our proposed algorithm covering the above procedures is illustrated in Fig. 1. For the computational calculation, we used Matlab® (The Mathworks, http://www.mathworks.com/products/matlab) for (1)–(4) and R packages for DEDS.

## Simulation study

**Fig. 2:**
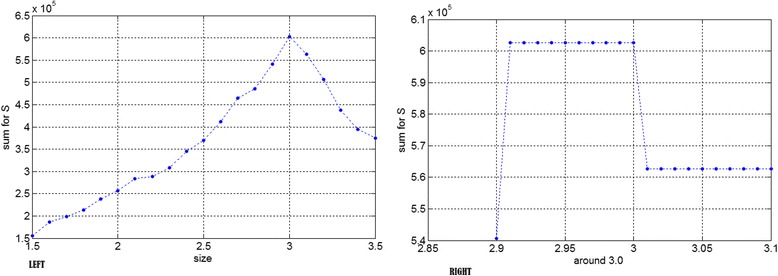
Plots for Fisher’s score and maximum point indicating the optimal threshold 3.0 (*left*). *Right panel* indicates plots of scores for more precise regions around 3.0

**Fig. 3:**
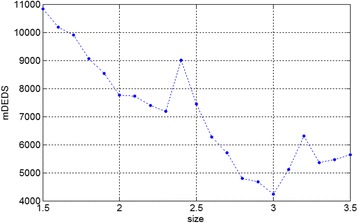
Plots for DEDS and minimum point indicating the optimal threshold 3

To confirm the accuracy of our proposed algorithm, we performed a simulation study. We considered three sets of artificial 10,000-array data, named ‘simdat1,’ ‘simdat2,’ and ‘simdat3.’ We first generated artificial data to represent tumor size. For the range of sizes, we generated random numbers by a uniform distribution between 1.0 and 2.9 and between 3.0 and 5.0, and thus the border size between small and large was set at 3.0. Simdat1 contains 55 small-sized samples and 45 large-sized samples, simdat2 contains 35 small-sized samples and 45 large-sized samples, and simdat3 contains 120 small-sized samples and 80 large-sized samples. Next, we generated artificial array data using random variables that follow different probability distribution functions to obtain higher and lower expression levels of the real data. Those higher and lower expressions are for the larger-size samples. Simdat1 was generated by a normal distribution with mean 10 and standard deviation 10 (described as *N*(10, 10)) as higher expression levels of 3500 arrays and *N*(−2, 10) as lower expression levels of 3500 arrays for 45 samples. The remaining array data within the expression levels other than those classified as higher or lower were generated by *N*(3, 1) for all samples. For simdat2, the higher expression levels with 2000 arrays were generated by a gamma distribution with shape 5 and scale 10 (described as *Γ*(5, 10)) and the lower expression levels with 4500 arrays were generated by *Γ*(3, 6) in 45 samples. The remaining array data were generated by *N*(0.5, 10). For simdat3, the higher expression levels with 2500 arrays were generated by a Poisson distribution with a parameter 10 (described as Pois(10)) and the lower expression levels with 3500 arrays were generated by Pois(8) in 80 samples. The remaining array data in 120 and 80 samples were generated by *N*(0.1, 20). These three datasets are illustrated in Additional file 1: Figure S1. Using a grid with difference equal to 0.1 within the range from 1.5 to 3.5, we estimated the optimal *q*_0_ satisfying Eqs. (1) and (2). Fisher’s scores for the range are illustrated in Fig. 2. The left panel indicates that the maximum point was at 3.0. The right panel shows the plots of the scores for 0.01 intervals between 2.9 and 3.1. Taken together, these results suggest that searching by Fisher’s score found the optimal threshold to be 3.0, with the greatest difference in expression level. Then mDEDS was applied, using all six *t*-, SAM, FC, *B*-, moderated *t*-, and moderated *F*-statistics. Figure 3 shows the plots for DEDS score according to the range and the minimum point indicating the optimal threshold 3.0.

**Fig. 4:**
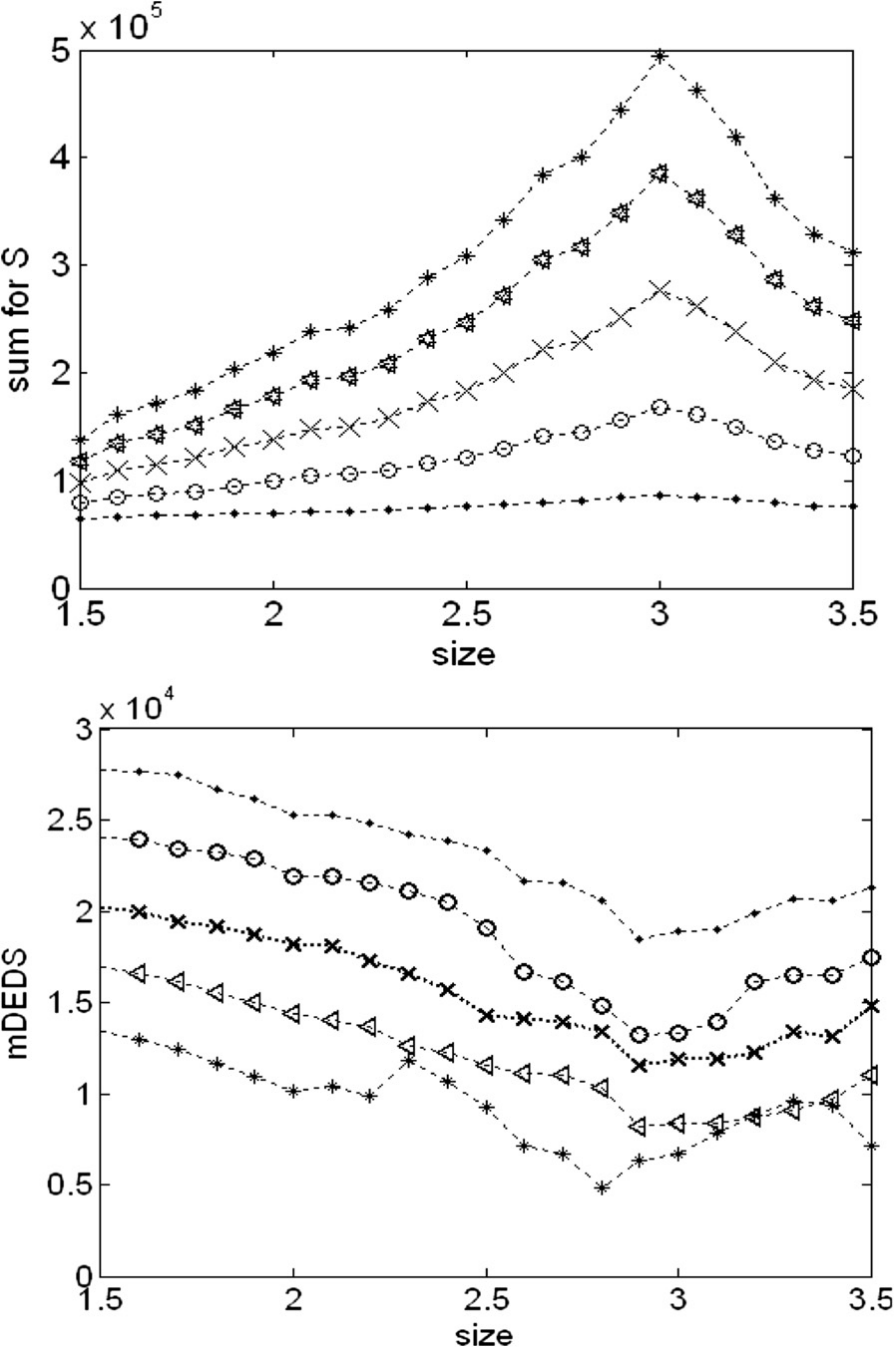
Plots for Fisher’s score (*upper*) and DEDS (*lower*) (*black small square* 5 %, *O* 20 %, *multiplication sign* 40 %, *triangle* 60 %, and *asterisk* 80 %)

To test how robust the proposed method is if a small portion of the features are DE, we also generated simulation data assuming the same statistical distributions but involving 5, 20, 40, 60, and 80 % DE genes. The upper and lower plots in Fig. 4 present the plots of the sum for S and mDEDS, respectively, with the different DE ratios. In the case of smaller difference in expression (5 %), the curves are flatter; however, the maximum for S showed an optimal threshold of 3.0 for each percentage of DE genes. The results for mDEDS appeared more unstable than those for S. When TEP was applied, the thresholds are summarized as 3.4 for 5 %, 3.1 for 20 %, 2.8 for 40 %, and 2.9 for the others. This suggests that TEP could show a more robust threshold for data at a higher DE percentage. In our breast cancer dataset, the percentage of DE genes is about 25 % in the largest case.

Summarizing these simulation studies, both Fisher’s and mDEDS scores found the optimal threshold 3.0, which was the boundary set for generating random small and large values. Thereby, we could demonstrate the validity of our proposed algorithm.

## Materials

### Norwegian datasets

Three datasets were gathered at two Norwegian hospitals. The two datasets consist of one-colored expression data (mdata1) (27 samples and 43,376 probes) and two-colored expression data (mdata2) (46 samples and 41,674 probes), which were collected at Akershus University Hospital, Lørenskog, Norway. The third dataset is 40,995 probes with one-colored mRNA expression for 102 tumor samples (mdata3), taken from patients with early-stage breast cancer [8] managed by Oslo University Hospital Radiumhospitalet in Norway. All datasets were processed on the Agilent platform, and the pre-processing of all datasets was performed by the methods provided by Bioconductor (http://www.bioconductor.org/help/workflows/oligo-arrays/). We applied quantile normalization to one-color data and the lowest normalization to two-color data. No background correction was performed for these data. The probes were matched across datasets. Consequently, 40,995 probes were used for the analysis. Given the relatively large full range of tumor sizes of 0.1–5.0 cm, however, the number of samples for less than 1.0 cm and over 4.0 cm were very few depending on the dataset. Therefore, we fixed 1.0–3.0 cm as the range we should search to find the optimum size.

### Validation datasets

To validate the optimum threshold estimated by the above datasets, we used the five different expression datasets, collectively called the Affy947 expression dataset [9]. The dataset is a collection of six published datasets containing microarray data of breast cancer samples. These datasets are all measured on Human Genome HG U133A Affymetrix arrays and normalized using the same protocol. Since one dataset (Pawitan et al. dataset [10]) did not involve the tumor sizes data, we excluded it from further analysis. They were assessable from NCBI’s Gene Expression Omnibus (GEO, http://www.ncbi.nlm.nih.gov/geo/) with the following identifies, GSE6532 for the Loi et al. dataset [11], GSE3494 [12] for Miller dataset, GSE7390 for the Desmedt et al. dataset [13], and GSE5327 for the Minn et al. dataset [14]. The Chin et al. [15] dataset is available from ArrayExpress (http://www.ebi.ac.uk/, identifier E-TABM-158). This pooled dataset was pre-processed and normalized as described in Zhao et al. [16]. Microarray quality-control assessment was carried out using the R AffyPLM package from the Bioconductor web site (http://www.bioconductor.org, [17]). The relative log expression (RLE) test and the Normalized Unscaled Standard Errors (NUSE) test were applied. Chip pseudo-images were produced to assess artifacts on arrays that did not pass the preceding quality-control tests. Selected arrays were normalized according to three-step procedures using the robust multi-array average (RMA) expression measure algorithm (http://www.bioconductor.org; [18]): RMA background correction convolution, median centering of each gene across arrays separately for each dataset and quantile normalization of all arrays. Gene mean centering has been shown to effectively remove many dataset-specific biases allowing effective integration of multiple datasets [19].

## Results and discussion

### Optimal tumor size

**Fig. 5:**
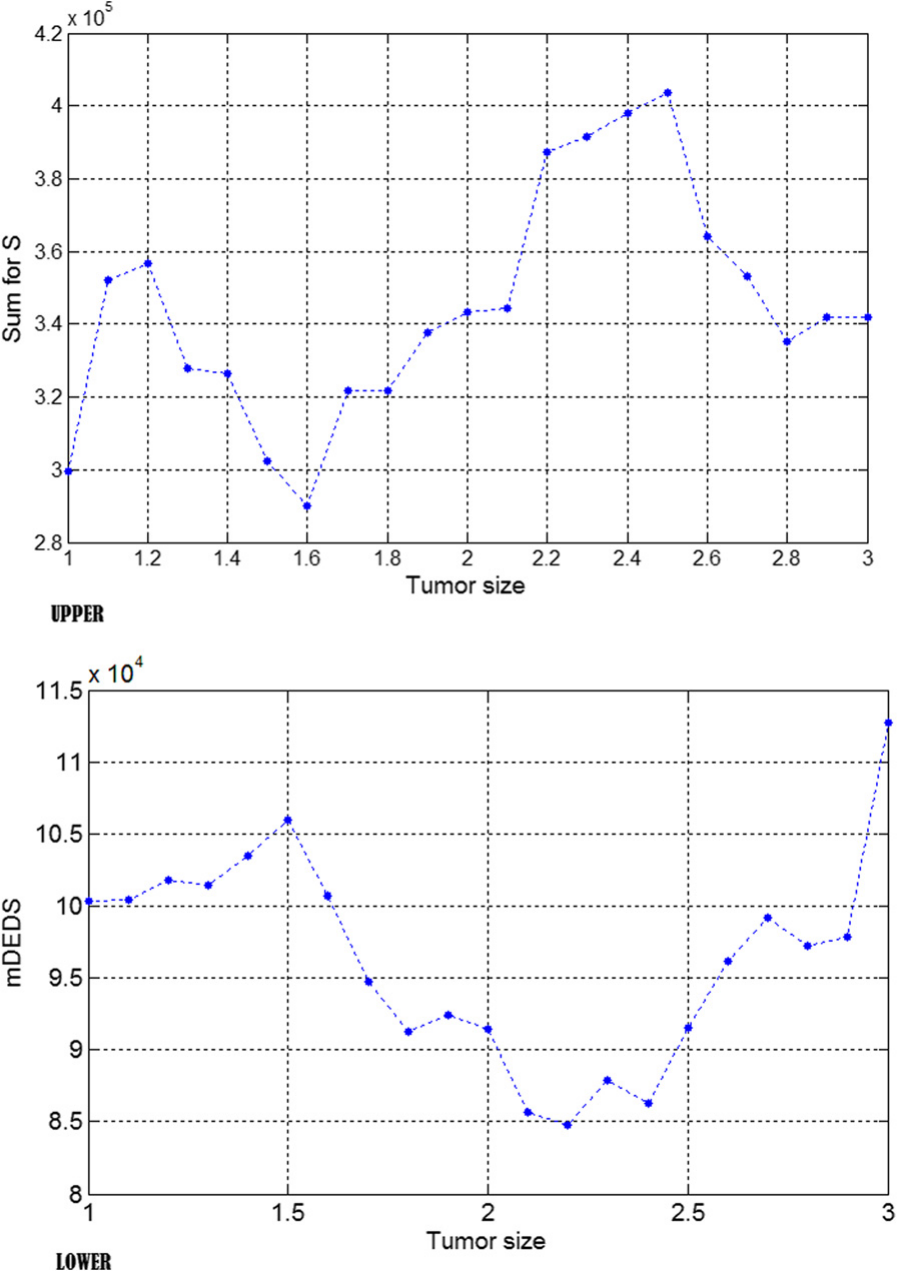
Fisher’s score (*upper*) found 2.5 cm and mDEDS (*lower*) found 2.2 cm as the optimal threshold

**Fig. 6:**
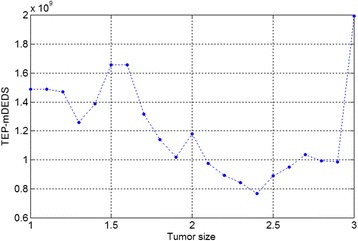
Plots obtained by TEP

Our proposed algorithm summarized in Fig. 1 was applied to the data across three different cohorts, and the plots for Fisher’s score and mDEDS are shown in Fig. 5. For mDEDS, we took all possible statistics, according to [6]: *t*-, SAM, FC, *B*-, moderated *t*-, and moderated *F*-statistics. Fisher’s scores estimated 2.5 cm as the optimal threshold, larger than the classical 2.0 cm. mDEDS determined 2.2 cm as the optimal threshold. For TEP, we summarize *q*_0 − TEP_ in Fig. 6. The minimum value for Eq. (4) was 2.4 cm, which was clearer than the result shown in Fig. 5 and closer to 2.5 cm obtained by Fisher’s score. This result suggests that TEP-based *q*_0 − TEP_ gives us a more robust threshold size. Given the results by Fisher’s score, it would not seem feasible to detect whether 2.2 or 2.4 cm is the best size. However, our proposed analysis can consider the possibility that a size larger than 2.0 cm is appropriate to indicate where the expression patterns show the greatest difference.

It is important to notice that the optimal value of *q*, obtained by optimizing the objective functions (3) and (4), cannot be equipped with a confidence interval obtained by bootstrap. This is similar to other situations in statistics, where certain parameters are obtained by optimization, for example, the smoothing parameter in non-parametric regression or the penalty in lasso regression, obtained by optimizing some cross validation criteria. To explain this, let us follow the bootstrapping paradigm. Let us fix a value *q*_1_. Then we can compute the *p* values *p*_*i*_(*q*_1_) and the Fisher score S*(*q*_1_). We can bootstrap the data and obtain bootstrap distributions for all *p* values and compute the corresponding bootstrap distribution for *S*(*q*_1_), which has a mean equal to S*(*q*_1_). We now repeat for various *q* in *Q* and obtain score S*(*q*) and the bootstrap distributions for score *S*(*q*) for all *q* in *Q*. What we do in this article is to minimize over *q* the score S*(*q*), which can be interpreted as the bootstrap mean. But we cannot minimize the sum of the bootstrapped distributions of *S*(*q*) for all *q* in *Q*. We need to summarize these distributions by a point estimate, and our method uses the mean. For example, we could use the bootstrap medians instead. In any case, the obtained optimal *q* cannot carry any bootstrap-based uncertainty. On the other hand, we can repeat the threshold selection separately on each of the three datasets. For mDEDS, this gave the optimal values of 2.1, 2.2, and 2.2 cm; for Fisher’s score, we obtained 1.7 (slightly preferable to 2.5 cm), 2.4, and 2.5 cm. Three values do not allow an estimate of variability, but they appear consistent.

### Validation study

**Fig. 7:**
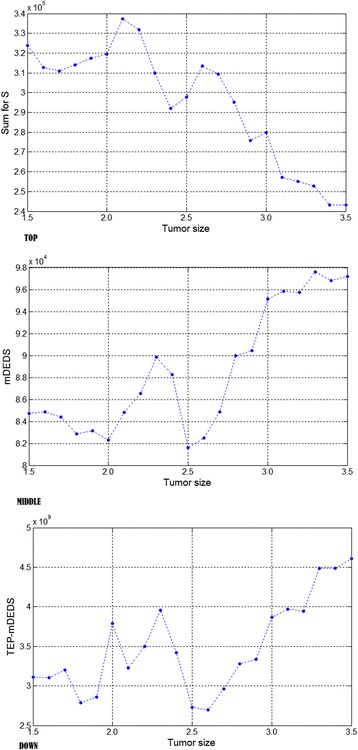
Optimum thresholds by Fisher’s score (*top*), mDEDS (*middle*), and TEP (*bottom*) in the case of validation datasets

To validate our proposed algorithm, additional five different expression data were analyzed using the same approaches. For mDEDS, we took six statistics, *t*-, SAM, FC, *B*-, moderated *t-*, and moderated *F*-statistics. The plots for Fisher’s score, mDEDS and TEP are shown in Fig. 7. Some studies involve few samples for smaller size than 1.5 cm or larger than 3.5 cm. Therefore, the plots should be shown within the range between 1.5 and 3.5 cm. The optimum sizes were 2.1 cm by Fisher’s scores, 2.5 cm by mDEDS, and 2.6 cm by TEP, which were all larger than 2.0 cm. If the first local maximum of 2.0 cm is ignored for the Fisher’s score, the second peak indicated 2.6 cm. These results suggest that the five datasets validate the possibility of a optimum threshold which is larger than 2.0 cm. On the other hand, the cases for mDEDS and TEP indicated 2.0 and 2.1 cm as the second peak. This confirmed that the 2.0-cm rule works for distinguishing different characteristics of the tumor in the expression data. Furthermore, we can say that the 2.0-cm rule is robust also with respect to the gene expression analysis, since it appears to be conservative in recommending a stronger treatment a couple of millimeter before a threshold based on the gene expression would indicate [2].

### Survival analysis using optimum threshold

**Fig. 8:**
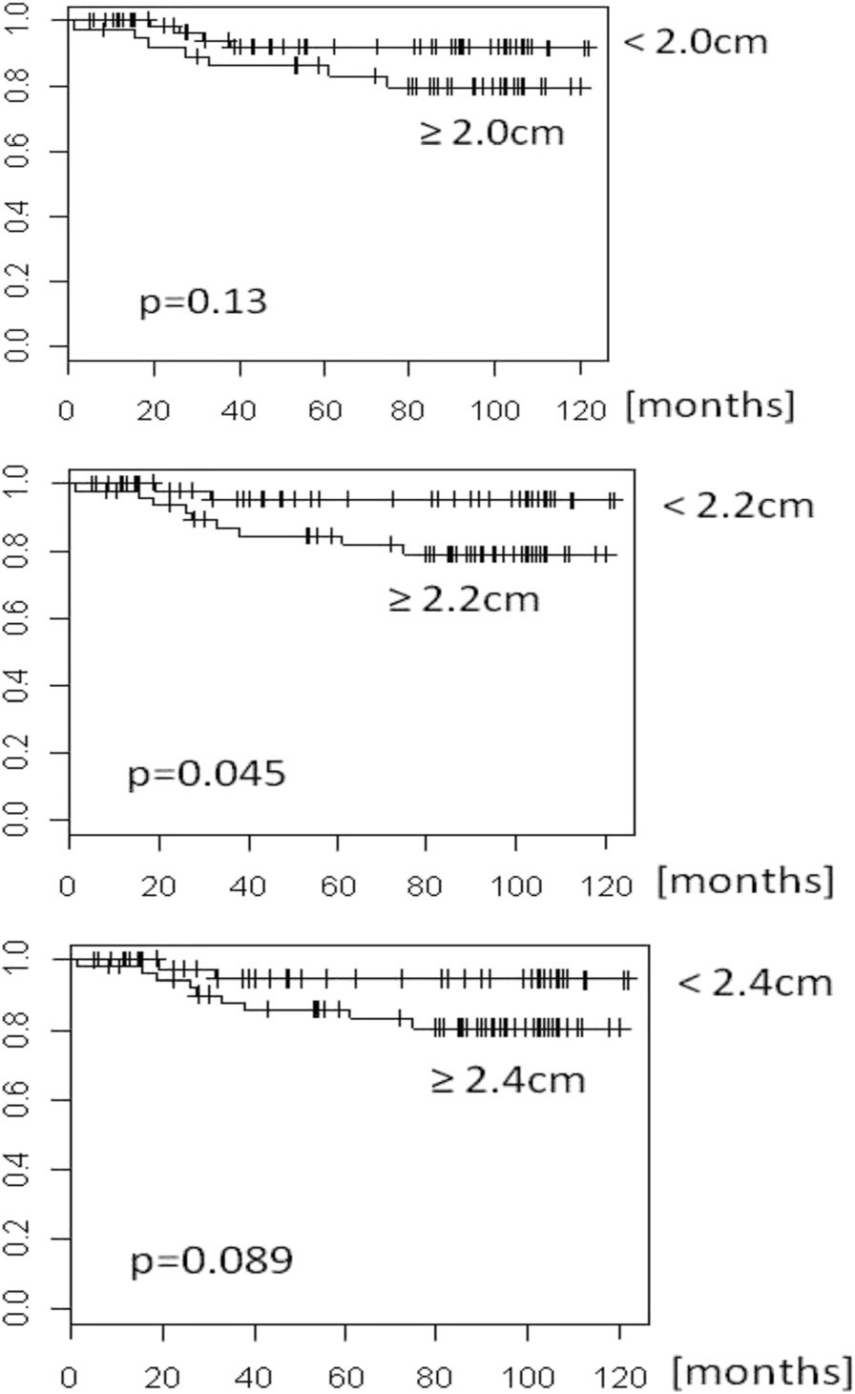
Survival curves in the case of local recurrence for each threshold 2.0 cm (*top*), 2.2 cm (*middle*), and 2.4 cm (*bottom*)

Usually, the goal for tumor staging based on tumor size and other factors is to guide the choice of treatment for patients and predict their outcomes. Therefore, we evaluate our threshold also with respect to clinical outcomes, namely the survival time and time to local recurrence. We have the monthly survival time (time to death) and time to local recurrence for only mdata3. We divided these patients into two classes according to the thresholds 2.0, 2.2, and 2.4 cm. The survival functions of the corresponding classes were compared by Kaplan-Meier analysis and the log-rank test. The survival was defined either as overall survival (death by any reasons used as the observed time and alive used as the censored time) or as breast cancer (BC)-specific survival (death by only BC used as the observed time and others used as the censored time). Table 1 summarizes the obtained *p* values for the log-rank test of each survival time and each threshold. The 2.0-cm threshold distinguishes best in terms of overall survival. Interestingly, the 2.0- and the 2.2-cm thresholds appear to be preferred in terms of BC-specific survival. The threshold 2.2 cm appears to provide the best classification for local recurrence. The survival curves of the two groups shown in Fig. 8 are more different for all thresholds larger than 2.0 cm. This result suggests that the optimum threshold, which maximizes the total differential expression also, is confirmed by the larger difference in time to local recurrence. Local recurrence is known to be better predicted by expression compared to overall survival. In summary, despite the limitations of our data, there is some indication that a slightly larger threshold between 2.0 and 2.2 cm, which maximizes differential expression, also leads to improved distinctions in survival curves for time to local recurrence, compared to the traditional 2.0-cm rule.

**Table 1:** *p* values obtained by the log-rank test for survival time (months) and time to local recurrence (in months)

Thresholds [cm]	Overall survival	BC specific	Local recurrence
2.0	0.021	0.027	0.13
2.2	0.18	0.047	0.045
2.4	0.30	0.083	0.089

### Associated network and canonical pathway analyses based on the gene lists of expression differences between T1 and T2 groups based on the 2.4- and 2.0-cm thresholds

We are interested in the specific biological features of the genes discriminating between tumors below and above the given 2.0- and 2.4-cm thresholds. First, we applied SAM [20] to obtain the significant probes in terms of gene expression differences for both thresholds. Table 2 summarizes the number of significant probes and the corresponding FDR [21].

**Table 2:** Estimated number of significant probes (genes) by SAM

Size used for response		2.4 cm		2.0 cm	
Statistics for SAM		# probes	FDR (%)	# probes	FDR (%)
Data	mdata1	12	15.03	44	6.03
	mdata2	11,740	5.02	8036	4.81
	mdata3	93	20.29	9	10.42

**Fig. 9:**
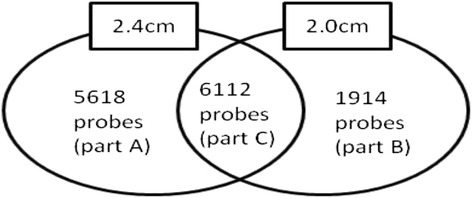
Summary of unique probes for 2.4 and 2.0 cm and overlapped probes

As shown in the table, since the two-color dataset (mdata2) keeps the 5 % FDR level, we focus on this dataset for the associate network and canonical pathway analysis. For the probes obtained by SAM, we counted unique significant probes for each threshold as well as the number of overlapping probes (see Additional file 2: Table S1). Figure 9 summarizes the numbers of unique probes—2.4 cm unique (part A), 2.0 cm unique (part B), and overlapped (part C)—in the Venn diagram. In order to investigate the biological functional interaction for the gene lists, we used a tool called IPA (Ingenuity Pathway Analysis) [22], which delivers a rapid assessment of the signaling and metabolic pathways, molecular networks, and biological processes that are most significantly perturbed in the dataset of interest. IPA has many options to find insights on the relationships, mechanisms, functions, and pathway of relevance. We selected an option for associated network functions and canonical pathway, and the outputs for the pathway analyses and biological functions (diseases and disorders, molecular and cellular functions) are summarized in Table 3. The *p* value associated with a biological process or pathway annotation is a measure of its statistical significance with respect to the Functions/Pathways/Lists Eligible molecules for the dataset and a reference set of molecules (which define the molecules that could possibly be Functions/Pathways/Lists Eligible). The *p* value is calculated with Benjamini and Hochberg FDR [21]. The ratio of the canonical pathways is defined as the number of molecules in a given pathway that meet the cutoff criteria divided by the total number of molecules that make up that pathway. Networks are scored based on the number of network-eligible molecules they contain. In Table 3, a score above 10 is recognized as a meaningfully higher score. The network score is based on the hypergeometric distribution (source: IPA online manual).

**Table 3:** Summary of pathway analyses

(Part A) Biological functions enriched in 5618 unique probes separating tumors below and above size 2.4 cm	
Associated network functions	Score
Cellular assembly and organization, cellular compromise, protein synthesis	26
Cell signaling, nucleic acid metabolism, small molecule biochemistry	24
Energy production, nucleic acid metabolism, small molecule biochemistry	24
Hair and skin development and function, dermatological diseases and conditions, developmental disorder	22
Post-translational modification, gene expression, infectious disease	22
Top canonical pathways	−log (FDR-corrected *p* value)
Neuropathic pain signaling in dorsal horn neurons	1.13
Role of NNFAT in cardiac hypertrophy	1.13
Melatonin signaling	6.94 × 10^−1^
Molecular mechanisms of cancer	6.94 × 10^−1^
Calcium-induced T lymphocyte apoptosis	6.94 × 10^−1^
Diseases and disorders	FDR-corrected *p* value
Cancer	3.95 × 10^−1^–5.44 × 10^−1^
Hematological disease	3.95 × 10^−1^–5.44 × 10^−1^
Immunological disease	5.13 × 10^−1^–5.44 × 10^−1^
Hypersensitivity response	5.44 × 10^−1^–5.44 × 10^−1^
Inflammatory response	5.44 × 10^−1^–5.44 × 10^−1^
Molecular and cellular functions	FDR-corrected *p* value
Gene expression	2.03 × 10^−1^–5.44 × 10^−1^
Cellular growth and proliferation	2.03 × 10^−1^–5.44 × 10^−1^
Energy production	2.03 × 10^−1^–5.44 × 10^−1^
Amino acid metabolism	2.03 × 10^−1^–5.44 × 10^−1^
Small molecule biochemistry	2.03 × 10^−1^–5.44 × 10^−1^
(Part B) Biological functions enriched in 1914 unique probes separating tumors below and above size 2.0 cm	
Associated network functions	Score
Antigen presentation, cellular movement, hematological system development and function	29
Cell assembly, and organization, cellular function and maintenance, protein synthesis	29
Gene expression, infectious disease, small molecule biochemistry	29
Cellular assembly and organization, cell signaling, gene expression	29
Post-translational modification, protein folding, cell death	24
Top canonical pathways	−log (FDR-corrected *p* value)
Tight junction signaling	9.13 × 10^−1^
Germ cell-Sertoli cell junction signaling	8.69 × 10^−1^
Cfc42 signaling	1.26 × 10^−1^
Fatty acid biosynthesis	8.69 × 10^−1^
Integrin signaling	8.69 × 10^−1^
Diseases and disorders	FDR-corrected *p* value
Dermatological diseases and conditions	6.58 × 10^−3^–2.73 × 10^−1^
Genetic disorder	6.58 × 10^−3^–2.73 × 10^−1^
Infectious disease	1.14 × 10^−2^–2.73 × 10^−1^
Inflammatory disease	3.38 × 10^−2^–2.73 × 10^−1^
Inflammatory response	3.38 × 10^−2^–2.73 × 10^−1^
Molecular and cellular functions	FDR-corrected *p* value
Antigen presentation	3.38 × 10^−2^–2.73 × 10^−1^
Cell-to-cell signaling and interaction	3.38 × 10^−2^–2.73 × 10^−1^
Cellular compromise	3.38 × 10^−2^–2.73 × 10^−1^
Cellular function and maintenance	3.38 × 10^−2^–2.73 × 10^−1^
Cellular movement	7.14 × 10^−2^–2.73 × 10^−1^
(Part C) Biological functions enriched in 6112 overlapping probes separating tumors below and above size 2.4 cm and 2.0 cm	
Associated network functions	Score
Protein synthesis, post-translational modification, cancer	26
Cell signaling, nucleic acid metabolism, small molecule biochemistry	24
Lipid metabolism, small molecule biochemistry, vitamin, and mineral metabolism	24
Connective tissue development and function, embryonic development, skeletal and muscular system development and function	24
Cancer, dematological diseases and conditions, tumor morphology	22
Top canonical pathways	−log (FDR-corrected *p* value)
Cytotoxic T lymphocyte-mediated apoptosis of target cells	3.65
Allograft rejection signaling	2.77
Nur77 signaling in T lymphocytes	2.77
Antigen presentation pathway	2.77
T helper cell differentiation	1.75
Diseases and disorders	FDR-corrected *p* value
Dermatological diseases and conditions	1.98 × 10^−7^–1.89 × 10^−1^
Respiratory disease	7.98 × 10^−5^–1.89 × 10^−1^
Cancer	4.49 × 10^−4^–1.89 × 10^−1^
Genetic disorder	4.49 × 10^−4^–1.71 × 10^−1^
Inflammatory response	4.49 × 10^−4^–1.89 × 10^−1^
Molecular and cellular functions	FDR-corrected *p* value
Cell-to-cell signaling and interaction	2.68 × 10^−4^–1.89 × 10^−1^
Cellular movement	8.02 × 10^−4^–1.89 × 10^−1^
Cellular growth and proliferation	1.29 × 10^−3^–1.89 × 10^−1^
Cellular development	2.99 × 10^−3^–1.89 × 10^−1^
Cell death	4.1 × 10^−3^–1.89 × 10^−1^

Associated network functions explain the tendencies of cellular assembly in tumor interaction for the early stage of tumors and energy production for the progressive stage of tumors. Part C represents a transitional stage from early to progressive, which involves associated network functions including lipid metabolism and cell signaling, nucleic acid metabolism, and small molecule biochemistry.

For the common genes shown in part C, besides known genes in breast cancer, such as *AKT*, *ERBB2*, and *PTEN*, we found also *MTDH*. When it was introduced, the gene *Metadherin* (*MTDH*) was shown to affect the expression of many genes of relevance to the metastatic and chemo-resistance phenotypes [23]. *MTDH* may also represent a novel mediator of malignant breast cancer progression. Furthermore, we found interesting genes in part A such as *MYC*, which is known as an oncogene frequently deregulated in breast cancer; *TP53*, which is associated with high risk for various cancers; *RAD50*, which is known to moderately increase breast cancer risk; and *BRCA2*, whose mutation is associated with a significantly elevated risk for breast and ovarian cancers [24].

## Conclusions

We study various tumor size thresholds that can be used to create two groups of patients. We proposed a numerical algorithm involving Fisher’s score and mDEDS using gene expressions. Both measurements found that the difference in gene expression between smaller and larger tumors appears to be slightly larger than 2.0 cm. The over 2.0-cm optimum thresholds were supported by a validation using the five published expression datasets. We also found that the thresholds over 2.0 cm lead to the most distinct Kaplan-Meier curves of time to local recurrence. From the associated network and canonical pathway analyses for Norwegian datasets, the lists of DE genes for the 2.4-cm threshold also included some genes related to the metastasis of breast cancer. The same approach can be extended to also controlling other factors such as tumor grades and estrogen receptor (ER) status, which are also important prognostic indicators for breast cancer. It could also apply to other cancer considering tumor size as a prognostic indicator. A further extension of our approach would be to determine more than two groups of patients, on the base of two (or more) thresholds. This would indicate that tumor dimension has a similar role with tumor grades. We decided to remain within the consolidated clinical practice with just the T1/T2 distinction. In summary, our analysis based on gene expressions indicates that the 2.0-cm rule applied to determine patients who will benefit from more aggressive therapy appears to be justified. However, we find indications that a slightly larger threshold, of 2.2 cm could instead be applied, thus reducing therapy for some borderline patients. This could spare negative effects of strong therapies to patients that possibly do not need them. We interpret our results as a call for a critical revision of the 2.0-cm rule in the light of individual genomic data.

## Additional files

Additional file 1: Figure S1. Simulated array data. Top: simdat1, middle: simdat2, and bottom: simdat3. (PDF 86.2 kb)
Additional file 2: Table S1. SAM's outputs for the unique and the overlapping significant probes. (XLS 1341.44 kb)

## Abbreviations

BC: breast cancer
DE: differential expression
DEDS: differential expression via distance synthesis
FC: fold change
GEO: Gene Expression Omnibus
MAD: median absolute deviation from the median
mDEDS: meta differential expression via distance synthesis
NCBI: National Center for Biotechnology Information
NUSE: normalized unscaled standard errors
RLE: relative log expression
RMA: robust multi-array average
SAM: significant analysis of microarray
TEP: totally extreme point
WMW: Wilcoxon-Mann-Whitney
