# Supplementary figures and images for: Gene expression analysis supports tumor threshold over 2.0 cm for T-category breast cancer
Source: EURASIP J Bioinform Syst Biol. 2016 Feb 8;2016:6. doi: 10.1186/s13637-015-0034-5 (PMC4746218; doi:10.1186/s13637-015-0034-5)

**Additional figure.**

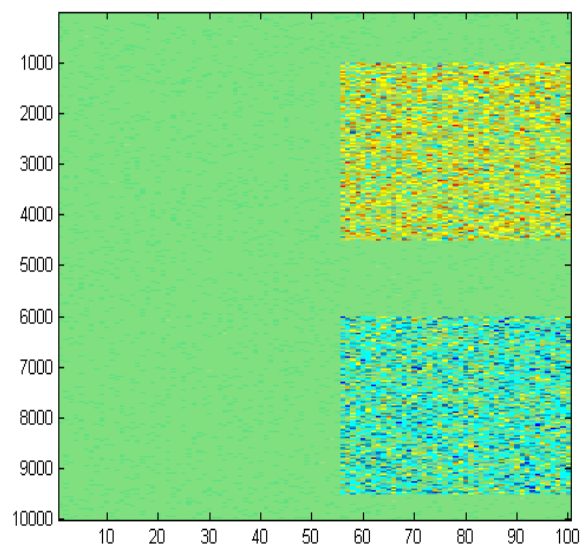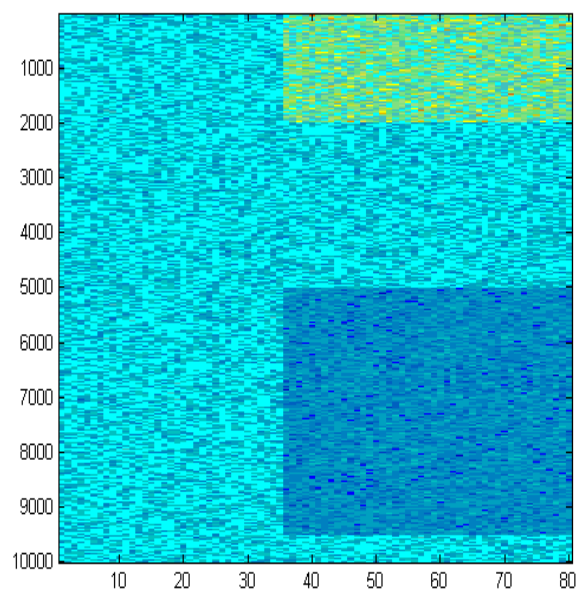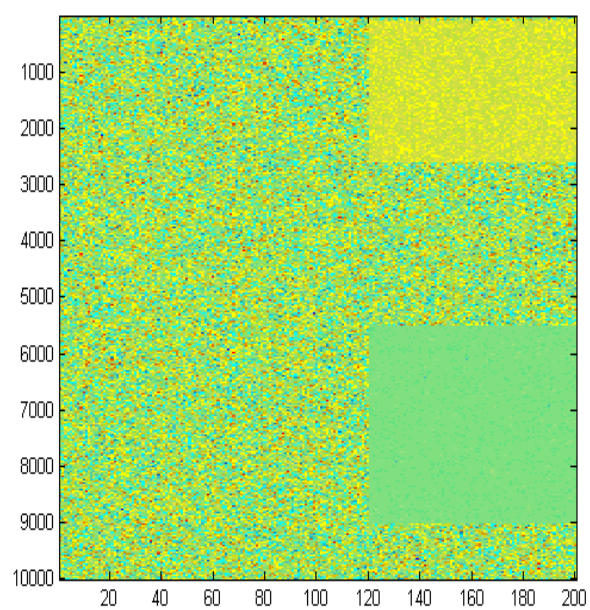

Supplement: Additional file 1: Figure S1. — Simulated array data. Top: simdat1, middle: simdat2, and bottom: simdat3. (PDF 86.2 kb) [file 13637_2015_34_MOESM1_ESM.pdf]
